# Commentary: Effect of acupuncture for poor ovarian response: a multicenter randomized controlled trial

**DOI:** 10.3389/fendo.2026.1838768

**Published:** 2026-05-14

**Authors:** Chenfei Wang, Xinmei Chen, Cong Wang, Renwen Wu

**Affiliations:** 1 Department of Acupuncture and Moxibustion, The First Affiliated Hospital of Zhejiang Chinese Medical University (Zhejiang Provincial Hospital of Chinese Medicine), Hangzhou, China; 2 Traditional Chinese Medicine Hospital Of QingYuan, Lishui, China

**Keywords:** acupuncture, dose-response, *in vitro* fertilization, methodological considerations, poor ovarian response, sham-controlled trial

We read with great interest the recent article by Su et al. reporting a multicenter randomized controlled trial evaluating acupuncture for poor ovarian response (POR) in women undergoing *in vitro* fertilization (IVF) treatment (1). Poor ovarian response remains a challenging and heterogeneous condition in assisted reproduction, and the Bologna criteria have provided one of the most widely used frameworks for its definition (2). This study is a valuable contribution to a field in which high-quality clinical evidence remains limited. In particular, the multicenter randomized design and the use of the number of oocytes retrieved as the primary endpoint enhance the clinical relevance of the study (1). However, several methodological and interpretive issues merit further discussion to better contextualize the findings and guide future research.

First, the absence of a sham acupuncture comparator substantially limits interpretation of acupuncture-specific effects. In the trial, participants in the acupuncture group received acupuncture pretreatment before controlled ovarian hyperstimulation (COH), whereas those in the control group received IVF alone without acupuncture pretreatment (1). Although this design allows evaluation of an “acupuncture pretreatment strategy” versus no acupuncture, it does not adequately distinguish specific effects of acupuncture needling from nonspecific influences, such as treatment expectation, differential patient engagement, and increased clinician contact. This issue is especially relevant in acupuncture trials, where sham-controlled designs and rigorous reporting standards are often recommended to improve internal validity and reduce performance bias (3, 4). Therefore, the current findings should be interpreted as evidence that acupuncture pretreatment was not superior to no acupuncture for the primary endpoint, rather than definitive evidence against acupuncture-specific biological effects (1).

Second, heterogeneity in actual treatment exposure may have diluted a potential dose-dependent effect. The protocol specified a relatively intensive acupuncture pretreatment schedule before COH; however, a considerable proportion of participants initiated COH early after completing only the minimum required treatment period (1). For interventions such as acupuncture, whose putative mechanisms may involve cumulative regulation of the hypothalamic-pituitary-ovarian axis, ovarian stromal microenvironment, or follicular development, insufficient and variable exposure may attenuate measurable benefit. This concern is particularly relevant because prior evidence suggests that the effectiveness of acupuncture in assisted reproduction may depend, at least in part, on treatment schedule and timing (5). More detailed reporting of the actual number of completed sessions, adherence distribution, and, if feasible, exploratory dose-response analyses would help readers determine whether the negative primary result reflects true lack of efficacy or underexposure to the intervention.

Third, the discordance between positive secondary signals and neutral reproductive endpoints warrants cautious interpretation. Su et al. found no significant between-group difference in the primary outcome, the number of oocytes retrieved, and also reported no statistically significant improvement in total clinical pregnancy rate or total live birth rate (1). By contrast, embryo cleavage rate and basal follicle-stimulating hormone (FSH) were significantly improved in the acupuncture group (1). While these secondary findings are interesting, their clinical significance remains uncertain because they did not translate into statistically significant gains in patient-important reproductive outcomes. In reproductive medicine, surrogate or intermediate markers should be interpreted carefully when live birth and clinical pregnancy remain unchanged. These results may therefore be better regarded as hypothesis-generatingsignals rather than confirmatory evidence of clinical benefit.

Finally, the study may have been underpowered for reproductive outcomes of greatest clinical importance, particularly live birth. The trial was designed around the number of oocytes retrieved as the primary endpoint, which is understandable from a mechanistic perspective; however, patients and clinicians are ultimately more concerned with pregnancy and live birth (1). A larger sham-controlled trial powered specifically for live birth-centered outcomes would therefore be valuable in clarifying whether acupuncture offers clinically meaningful benefit in selected POR subgroups.

In summary, this multicenter randomized trial provides important evidence regarding acupuncture pretreatment for POR, but several issues deserve closer consideration, including the lack of a sham comparator, variability in treatment exposure, and the discrepancy between surrogate improvements and neutral reproductive endpoints. We believe that these considerations may help readers interpret the findings more cautiously and may also inform the design of future trials in this challenging population.
